# Understanding the sustainability debate on forest biomass for energy in Europe: A discourse analysis

**DOI:** 10.1371/journal.pone.0246873

**Published:** 2021-02-17

**Authors:** Zachary James Mather-Gratton, Søren Larsen, Niclas Scott Bentsen

**Affiliations:** 1 Faculty for Forest Science and Forest Ecology, Georg-August University, Göttingen, Germany; 2 Department of Geosciences and Natural Resource Management, University of Copenhagen, Frederiksberg, Denmark; 3 Danish Energy, Frederiksberg, Denmark; University of Georgia, UNITED STATES

## Abstract

The legislative process before the adoption of the revised European Union renewable energy directive mobilised various actors around the forest biomass issue in Europe. Which storylines do actors use to discuss and define the sustainability of forest biomass, how are the differences between the existing storylines explained, and can distinct ‘discourse coalitions’ of actors be observed as following each storyline? These questions are addressed through a discourse analysis to critically evaluate the debate around the utilisation of forest biomass for European renewable energy to identify persistent storylines adopted by discourse coalitions as they communicate their understanding of the issue, and compete to influence the policymaking and public perception. The hypotheses are that there are more than the hypothetical binary arrangement of pro versus anti storylines, and that some actors follow multiple storylines. Locating the methodological approach on the two dimensions; text versus context and critical versus constructivist, this study pays closer attention to context rather than on individual linguistic elements of texts. Regarding the second dimension, this study builds upon constructivist epistemology, being concerned with understanding which truths these storylines produce for their speakers, and their external influences upon alternative storylines and actors. The three storylines presented here represent three competing discourses regarding forest biomass usage in European renewable energy: forestry prioritised, climate focussed and critical. Each of these are promoted by actors aiming to gain discursive hegemony on the issue, both in terms of the impact of their discourse upon EU policy making and in the eyes of the public. Despite the discursive differences created by these deeply held opposing views of what sustainability and nature are and what this means for forest biomass, there were several points where narrative elements overlapped. These can provide insight for developing a more constructive debate on the sustainability of forest biomass.

## Introduction

### EU forest biomass policy

For decades, biomass has consistently provided around two thirds of annual European renewable energy production [1]. Biomass from forest sources (referred to here as ‘forest biomass’) provides the major part of this, in 2017 for example providing 69% of overall biomass [2]. Biomass is projected to continue to provide a significant portion of renewable energy (50% or more) for the decade to 2030, for electricity and in particular for heat due to its capacity for inclusion in domestic heating systems as well as meeting the higher energy needs of industry [3–5].

Optimism surrounded the EU’s initial adoption of biomass within renewable energy policy [6], with multiple touted benefits including energy system diversification, greenhouse gas (GHG) emission reduction, rural employment, decreased oil prices, and furthering the *“EU’s technological leadership”*. *“These benefits can be expected to be obtained without additional pollution or other forms of environmental damage”* [6 p 6]. Cross-compliance with existing EU regulations on agriculture, forestry, and land use were expected to ensure sustainable biomass energy consumption, however the inadequacy of this design quickly became apparent in terms of ensuring biomass delivers climate benefit while minimising associated environmental and socio-economic risks [7]. In December 2018, the revised renewable energy directive (REDII, Directive 2018/2001) [8] entered into force, as part of the Clean energy for all Europeans package and with the aim of helping the EU meet its commitments under the Paris Agreement. The new directive established a new binding renewable energy target for the EU for 2030 of at least 32%. While the RED (Renewable Energy Directive: Directive 2009/28/EC) [9] originally focused on environmental sustainability of liquid biofuels, the REDII introduced sustainability criteria for forest biomass and GHG savings thresholds for solid and gaseous biomass fuels. Along with the optimism by which forest biomass was initially included in EU renewable energy policy [6], a complex array of sourcing and usage scenarios explains the weaknesses in its regulation to date relative to other bioenergy types [10].

The environmental and social costs and benefits of using forest biomass vary widely depending on multiple interrelating factors, including the scale considered, and the type and source of biomass used [11–13]. The scientific literature reflects this: it is possible to find peer reviewed papers supporting most arguments along the pro-anti biomass spectrum [14]. This uncertainty and complexity has stimulated intense debate between actors competing for influence on forest biomass regulation [10, 15, 16]. At the heart of the argument are disagreements regarding the concepts carbon neutrality and carbon debt, and whether sustainable forest biomass utilisation can be guaranteed. Institutional conventions in bioenergy emissions accounting have led to forest biomass becoming normatively defined by many actors as carbon neutral [17]. This assumption continues to shape legislation and perceptions regarding forest biomass in renewable energy systems.

Critics however call upon time as a missing but crucial factor. Terrestrial carbon storage and sequestration rates are reduced following harvests, while combustion immediately emits CO_2_ [12]. Therefore, a carbon debt exists as it potentially takes decades or even centuries for forest regrowth to sequester equivalent CO_2_ to that emitted by harvesting and combustion [15]. Calculating carbon debt is complicated because it requires a knowledge of the forest carbon baseline in the counterfactual scenario where bioenergy harvest did not take place, as well as an accurate accounting of fossil fuel displacement [12, 18]. Apart from the temporal dimension, there is a spatial dimension to the debate on forest biomass utilisation. The use of forest biomass is linked to land use and land use change and the potentials for conflict are location specific. Industrial forestry is found to be in conflict with reindeer herding in Northern Sweden [19], and stump extraction for bioenergy in Finland raised debate over climate impacts versus other environmental impacts [20], demonstrating that the wider debate on forest bioenergy at EU level hides local debates spurred by local practices, traditions and conditions.

Sustainability criteria are a key mechanism proposed and currently used to ensure that forest biomass sourcing and utilisation meet agreed standards to avoid climate, environment and societal harm [21, 22]. While some member states have voluntarily initiated standards, the development of EU wide criteria within RED II was another source of controversy. van Dam, Junginger [23] describe the various proposals emerging from international organisations, governments, industry stakeholders and NGOs. These criteria target climate, environmental and socio-economic impacts, with the prioritisation of each depending on the aims of the proposing organisation. While forest biomass industries have generally been highly receptive to the development of sustainability criteria, many NGOs remain sceptical about their ability to fully mitigate risks to climate, environment and society [10, 24, 25]. As with many environmental problems, the forest biomass issue can be considered an inter-discursive amalgamation of multiple discourses [26, 27], drawing on a diverse array of knowledge by encompassing fields including forest ecology, silviculture, energy systems, climate science, economics and philosophy. Understanding each of these discourses requires technical specialism making it difficult for any single actor to understand the issue in full.

### Sustainability perceptions

Differing opinions regarding the sustainability of forest biomass indicate that a closer look at the term and its usage in relation to the debate is worthwhile. In Hajer’s [26] study he posited that ecological modernisation, at the time an emerging concept based on the assumption that global environmental catastrophe is best avoided by advancing global growth and technological capacity [28], would emerge as the dominant ideology in environmental policy making. Seghezzo [29] confirms this prediction. He raises further points where institutional interpretations of sustainability and the sustainable development paradigm as introduced by the 1987 World Commission on Environment and Development (WCED) Brundtland report [30], are contested in social science and by environmental actors. The term itself, *sustainable development*, indicates that the environmentalists’ cry for a return to a society in which economic growth and technology are no longer prioritised is off the table [31]. Instead an anthropocentric view of our relationship with the world guides an approach where mitigating climate change and other environmental problems will require achieving further control over nature and embracing technological advancement to sustain human societies. The variable treatment of natural resources within the sustainable development concept has been classified by the terms ‘weak’ and ‘strong’ sustainability [32]. In the former, the anthropogenic benefits gained from natural and manufactured (non-natural) capital are deemed equal and fully substitutable. Strong sustainability on the other hand rejects this. While the consumption or destruction of manufactured capital is generally reversible, natural resources are a product of complex and irreplaceable interacting natural systems [33]. The forest biomass issue is placed squarely between these opposing visions of weak versus strong sustainability.

Forest management alone relates to several salient issues including climate change mitigation, biodiversity conservation and environmental restoration. With the preferred way in which forests should be managed in response to each of these shaped by actor values and experiences, there clearly exists an array of opportunities for diverging views and disagreement regarding forest biomass utilisation for energy. The role of science within the forest biomass debate becomes a key element to examine, particularly in relation to nature and sustainability concepts.

Discourse analysis provides a useful tool for determining how communication is used to establish a particular version of the world against competition from alternatives [34]. Hajer [26] describes the role of ‘storylines’, which function to reduce the discursive complexity of a problem, provide coherence and some permanence to an otherwise disparate array of discourses, and enable different actors to expand their understanding of a problem beyond their expertise while observing how their work relates to a wider picture. Within Hajer’s storyline concept, ‘discourse coalitions’ are loosely tied groups of actors such as organisations, companies, academics, politicians, or activists, formed of those prescribing to a particular storyline. Such coalitions are unconventional groups; the individual actors may not have met or discussed any strategy. However, the result of their shared mobilisation is a common understanding and communication of a given issue via a shared storyline [26].

### Aim

This study undertakes a discourse analysis to critically evaluate the debate around the utilisation of forest biomass for European renewable energy, using Hajer’s [26] approach to identify persistent storylines adopted by discourse coalitions as they communicate their understanding of the issue, and compete to influence the policymaking and public perception. The following questions were addressed: Which storylines do actors use to discuss the use and prospects of forest biomass for energy in the EU and how are the differences between the existing storylines explained? The hypothesis is that there are more than the hypothetical binary arrangement of pro-anti storylines, and that differing applications of three key concepts are driving the different storylines, these are: definitions of sustainability and whether it favours economic, ecological or social aspects; definitions of nature, and how it relates to forest management goals, and; the use of science and expert knowledge.

Can distinct ‘discourse coalitions’ of actors be observed as following each storyline? The hypothesis is that some actors follow multiple storylines.

## Theory and methods

### Discourse and discourse analysis

Phillips and Hardy [35 p 3] define discourse as *“an interrelated set of texts*, *and the practices of their production*, *dissemination*, *and reception”*. Their definition of texts includes any discursive unit, including speech, writing, images, and even objects. The present study considers writing as well as the speech and visual elements present within video media as research material. In this ontological approach, discourse creates meaning not via individual texts in isolation, but via interactions between texts and their contexts (including their production, distribution and consumption), as well as with other discourses [35, 36]. Thus Healy [37 p 293] defines discourse as a suite of *“dynamic ‘relational complexes’ involving people*, *things and their many properties*, *competences and accomplishments*.*”* These definitions are based upon strongly constructivist assumptions, which consider social structures and the meanings and categories used to define the world as continually constructed and revised by social interactions [38]. These discourses then are a product of the social context in which they are located and are connected with a multitude of further interrelated discourses.

**Fig 1:**
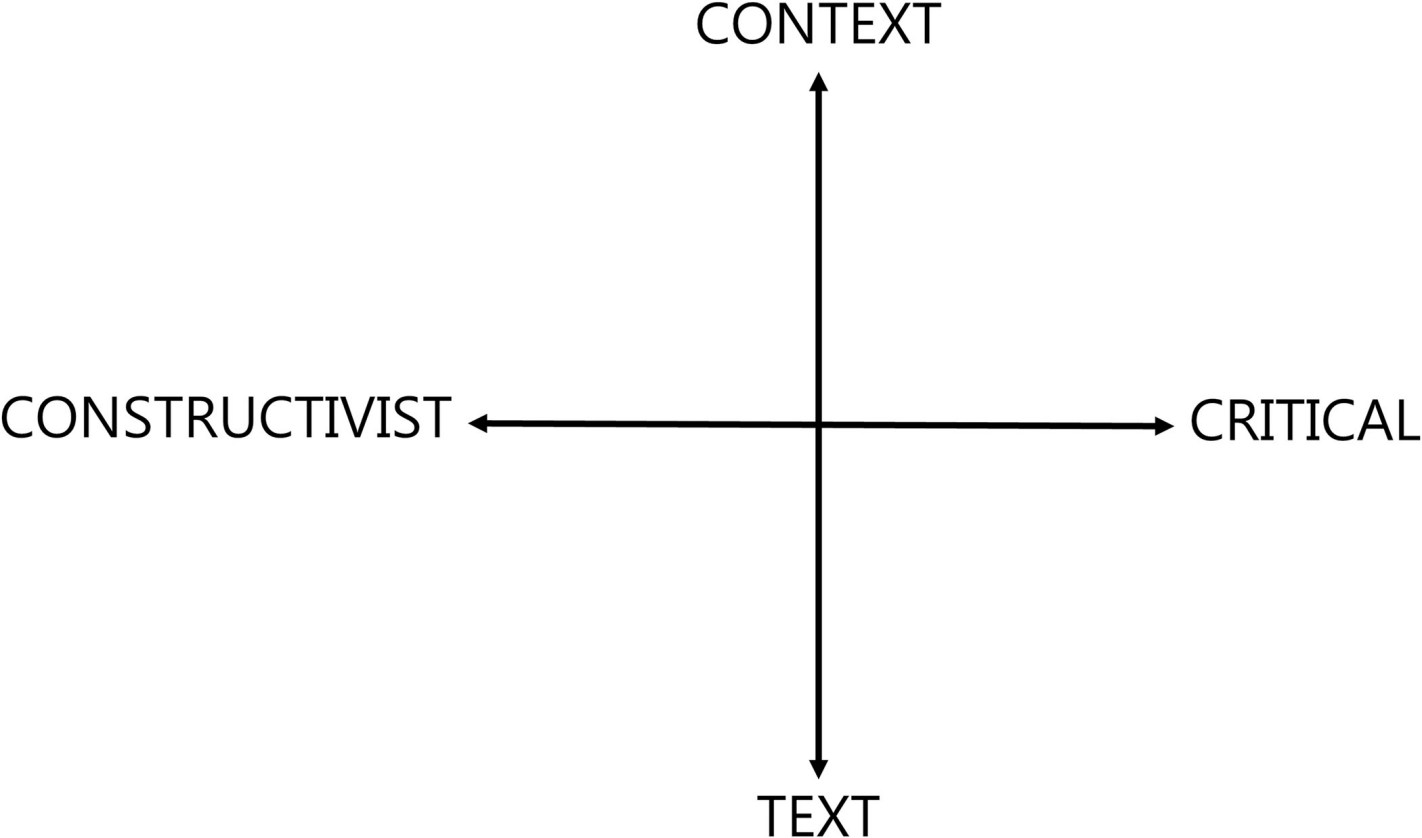
The different approaches to discourse analysis, modified from the version presented by Phillips and Hardy [35].

Discourse analysis is a methodological approach using the study of texts (using the above definition) to determine the constructive effects of discourse, along with the contextual relationships between texts and other discourses termed by Fairclough [36] as ‘interdiscursive’ factors. It also embodies a particular perspective to qualitative research, in which discourse becomes an active element in shaping social practices [39]. Discourse analysis therefore provides a useful tool for investigating the interrelationships of context upon the language by which environmental problems are debated, and vice versa. Phillips and Hardy [35] provide an overview of the epistemological and methodological characteristics of the different discourse analysis styles, presented on framework of two dimensions as shown by Fig 1.

The first dimension (the vertical axis on Fig 1) considers whether more attention is paid to a detailed analysis of individual texts, or to the wider contexts in which they are located. Given the concern of discourse analysts with the social construction of language, studies will necessarily consider both individual texts and their contexts [40]. The second dimension describes the degree to which the analysis is concerned with power and ideology as fundamental to the discourse process (a more critical approach), or whether the focus is more constructivist, concerning the social construction of meaning and reality through discourse.

### Analysis, scope and coding system

The legislative process before the adoption of RED II in 2018 has mobilised various actors around the forest biomass issue in Europe, both within and external to the political system. Discourse analysis was selected as an appropriate tool for investigating the terms of this dynamic debate. This study takes a broad view of the storylines and constituent narratives formed by the actors involved. Locating this methodological approach on the two dimensions presented on Fig 1, this study pays closer attention to context; the interrelationships between texts and external discourses, rather than the individual linguistic elements of texts. This broad approach was also adopted in recent discourse analyses studies of biomass energy in mass media [41, 42], indicating that the method is appropriate for unpicking the forest biomass debate, even if other studies have chosen other methodologies such as stakeholder surveys and interviews [43]. Regarding Phillips and Hardy’s [35] second dimension, this study is built upon a constructivist epistemology, meaning that the investigation is concerned with understanding which truths these storylines produce for their speakers, and their external influences upon alternative storylines and actors. Following Arts, Appelstrand [44] this study adopts a thin discourse analysis approach. The identified discourses are of course assumed to influence policy development, but we do not assume all policy making to be driven solely by the discourses analysed here. Identifying additional discourses and external influential factors is outside the scope of this study. To be noted also is that this study will not capture the *entirety* of the forest biomass discourse as only specific actors and texts are included. A broader discourse exists around forest biomass outside of the scope of this study, for example in government and academic circles.

### Actors and literature

The actors considered in this study are those located outside formal EU policymaking channels but attempting to shape it via their influence. This includes major industrial interests in the energy and forestry sector, scientists and academics, and environmental NGOs. Peer reviewed scientific literature was not included during the text gathering process as the research focus is upon the discourse formed by texts in the publicly accessed domain. However, given the specific focus of this research upon the role of science within the competing discourses around forest biomass, the analysis necessarily included an examination of any scientific literature included as S1 File for the narratives presented within the texts.

The literature search was limited to the last decade, 2008–2018 up until the adoption of REDII in December of 2018. This period coincides with the initial inclusion of bioenergy and biomass legislation within the 2009 EU renewable energy directive [9], and the consultation, advocacy and lobbying processes surrounding the revision of these legislatures in REDII.

The source texts included: reports; articles published on organisation websites, in online trade magazines and on blogs; position papers; press releases; open letters; and video media. In total 51 texts were gathered in the following means. Firstly, Internet (Google) searches were employed using combinations of the terms “biomass energy / bioenergy” with “Europe / European / EU / RED”. This also revealed several actors for inclusion in the analysis, and a snowball method was employed where source texts revealed further actors and texts to include in the investigation. The second literature gathering process was conducted by searching specific actor websites to identify relevant documents. Several actors provide comprehensive libraries of their documents, including reports, press releases, and open letters. In some cases, these libraries also include documents from or published in collaboration with other actors. The organisations AEBIOM (now called Bioenergy Europe), Biofuelwatch, FERN and Greenpeace, all provide such libraries, which were mined extensively for relevant texts. The results of these searches were manually filtered for content relevance. For inclusion, texts were required to refer explicitly to forest biomass utilisation for energy in an EU context (i.e. not solely within one individual EU member state). Specific reference to the RED was not required. This approach also resulted in an exclusion of texts from various actors, e.g. forest owners and forest product industries because they did not publish texts covered by our inclusion criteria that focus mainly on bioenergy/biomass energy. A full list of source texts is presented as S1 File.

### Coding process

The coding process was conducted via the following protocol. After an initial scan to determine the relevance of the text as described above, included texts were copied into a table breaking down the text by paragraph into each row. Several close reads were conducted, and notes and highlights were added to the texts to identify specific themes discussed in the text, such as sustainability, forest management, human health and climate change. Notes were then made regarding how these themes were presented in terms of the language used, the framing applied to certain issues. Where key issues were not discussed, this too was included in our analysis, for example if concurrent texts from multiple actors referred to a particular event or topic, the content itself and what was left unsaid becomes highly relevant. The results of these individual coding sessions for each text were collated, enabling comparison between texts towards their treatment of the specific themes. This then enabled the identification of specific storylines formed by the texts.

## Results and discussion

### Storylines around forest biomass

Our interpretation revealed three forest biomass storylines presented by the gathered texts. These align along a rough continuum in terms of their support for forest biomass and their dominant narratives regarding the issue’s numerous points of controversy. We named the storylines as follows: *forestry prioritised*, *climate focussed*, *and critical*. These are referred to as storylines 1, 2 and 3 in the text.

A recent Dutch study [43] found five “perspectives” through a survey, interviews and meetings and investigated the arguments relating to 10 topics, e.g. climate, land use, energy transition, economy etc. Several of these topics correspond to the storyline elements of our analysis. Then the identified arguments were divided into a dichotomy with regards to using arguments which emphasize opportunities or risks of using bioenergy, i.e. “for or against” so to speak. This analysis showed that it is difficult to group stakeholders into supporters and opponents and that five “perspectives” emerge that can explain the position in the debate.

These storylines are presented here in turn, after which follows a deeper theoretical discussion of what their competing discourses mean in relation to the presented research questions, the relationships between the storylines, and their implications for policy making in the context of EU RED II. Table 1 presents a comparison of each storyline’s treatment of key narrative elements.

**Table 1:** Comparative summary of storyline elements.

Storyline element	Storyline 1: Forestry focussed	Storyline 2: Climate focussed	Storyline 3: Critical
**Carbon neutrality**	Forest biomass is a carbon neutral energy source	Is overly simplistic and instead a detailed case by case basis should be employed	Is an incorrect assumption based on false climate impact accounting
**C cycle time scale and climate**	Is unimportant as cumulative CO_2_ emissions are reduced in the long term	Regulations must ensure short term benefit to climate	Large-scale biomass always harms the climate
**Forests are expanding**	This additional wood volume should be utilised. These forests also require further management	Because they are recovering from centuries of over harvesting.	-
		This is an opportunity for climate change mitigation via storage	
**Forest management**	Good forest management through SFM practices will ensure sustainable biomass	SFM goals and renewable energy don’t need to be combined in legislation	SFM goals and other sourcing criteria are insufficient and open to manipulation, representing greenwashing
**Which biomass can be used?**	All biomass, regulated by markets	That which an LCA determines will provide climate benefit. Not “whole trees”, but restricted to wastes and residues	None to be supported by the EU RED. Potentially small-scale local schemes
**On progress and the bioeconomy**	Forest management is an integral part of the bioeconomy and forests should be fully exploited as capital	Forests should be managed to maximise the ecosystem services they provide	The technocratic assumptions and growth based goals of the bioeconomy concept are dangerous
**Ecological impacts**	Are manageable through regulation or certification. SFM rules govern good practice, and forests and biodiversity benefit from management	Current and proposed sourcing presents several major risks to biodiversity worldwide, and regulation and certification may be weakly enforced.	The current and future level of biomass utilisation crosses numerous ecological limits even when certified. Therefore biomass should not be defined as renewable
**Social impacts**	Benefits rural communities through job creation	Climate and environment given priority. Threats and opportunities exist for society	Major threat to societies worldwide
**Global impacts**	Negligible: sourcing is mostly “local” from EU forests. Imports are well regulated	Europe is setting a global bad example: meeting renewable energy targets with forest biomass here will encourage others to follow	Matches storyline 2, plus major impacts on the ‘global south’
**Human health**	-	Not specifically addressed but could be added in future	Combustion exerts major unavoidable impacts on health
**Natural capital**	Forest ecosystems are cultivated natural capital	Forest Ecosystems are critical natural capital, but can be monetized in a ecosystem services concept	Rejects natural capital. Forest ecosystems are beyond valuation in monetary terms

### Storyline 1: Forestry prioritised

This storyline presents forest biomass utilisation as a multiple-win opportunity, in which sustainable forest management, environmental sustainability, economic gain (including rural community viability) and climate change mitigation are mutually achievable elements, although some differences emerge between actors and texts regarding the priority assigned to each [45]. These proposed co-benefits depend upon three key assumptions: that forest biomass is a carbon neutral energy source, that an ideal forest exists and requires sustainable forest management to achieve, and a technocratic definition of biomass as a resource. The foundations of these assumptions are expanded upon below. Ter-Mikaelian, Colombo [17] describe the persistence of the carbon neutrality assumption, which remains prominent in biomass industry actor discourses as well as influencing policy decisions [10] despite a growing number of studies finding that the rationale is overly simplistic, particularly from a climate change mitigation point of view [11, 46, 47]. Hajer’s [26] storyline concept is useful here in explaining this persistence. An assumption that forest biomass is carbon neutral forms the foundation of all texts in this storyline, whether explicitly stated or strongly implied, representing a universally adopted baseline argument upon which to advocate less risk averse biomass utilisation approaches.

#### Carbon neutrality

Searchinger, Hamburg [46] describe how this neutrality assumption within EU and global climate change mitigation policy emerged from flaws in Kyoto Protocol and IPCC carbon accounting procedures for bioenergy and land use. This may explain the roots of the assumption within storyline 1 and its persistence. However, the normative influence of these institutional accounting rules was not revealed in the analysed texts. Instead, the analysis revealed that the carbon neutrality concept is described by storyline 1’s proponents in one of two subtly different ways, both relating to forest ecosystem carbon cycling. In the first, the emissions from forest biomass is classed as carbon neutral due to equivalent forest growth and carbon sequestration on a landscape scale [48, 49]. Tony Juniper is a former Friends of the Earth (FoE) UK chairman enlisted by Drax as an advisor via his environmental consultancy firm, Robertsbridge. Since this appointment, Juniper has published regular online magazine articles and appeared in videos promoting the sustainability of Drax’s biomass operations. *“I saw how*, *at the level of the landscape*, *there is no carbon debt”*, says Juniper [49]. *“Working forests absorb more C than they release”*, and therefore if we take biomass from *“forests that are growing”*, then any climate impact can be assumed negligible. In the second, forest biomass is presented as carbon neutral because it has already sequestered an equivalent quantity of carbon during its historical growth [50–52]. At the heart of this assumption that biomass is benign or beneficial from a climate point of view is a perception that fossil fuel and plant-based CO_2_ emissions are fundamentally different, given the much shorter C cycling rate (decades-centuries) of living vegetation relative to fossil fuel sources (millennia-millions of years) [53]. According to this storyline, there is a distinction between the two, often explained by diagrams illustrating their respective cyclical versus linear natures [54].

#### Time

Table 1 shows, the storylines differ in whether they emphasise short- or long-term climate impacts of forest biomass utilisation. The proponents of storyline 1 draw special attention to the strong relationship between cumulative CO_2_ emissions and climate change presented by the IPCC [55]. This is used to argue that *“the exact timing of CO*_*2*_ *emissions is less important than how much carbon is emitted in total”* [51 p 3]. The framing choices made by storyline 1 regarding this timing issue becomes apparent after a closer look at the same IPCC report which advocates *“substantial cuts in GHG emissions over the next few decades”* [55 p 77], a message of urgency also called for in wider climate change mitigation discourses but absent in storyline 1. Above all storyline 1 states that the mitigation benefits provided by forest biomass utilisation for energy are high, a view neatly expressed by EUSTAFOR, CEPF [56 p 5] *“Increasing the share of renewable sources of energy*, *such as biomass from forests*, *is consistent with reducing greenhouse gas emissions*.*”*

#### Scale and impacts

Whether discussing forest biomass sourced in Europe or worldwide, the story uses scale in different ways depending on the potential impact being discussed. When describing climate mitigation benefits, texts routinely define forest biomass as *“one of the main drivers of Europe’s energy transition”* [57 p 2], pointing to the large percentage of Europe’s renewable energy consumption it provides. The high percentage of the RED targets which biomass currently fills is used here to reinforce its importance in continuing to meet these targets as a *“clean*” form of renewable energy [58 p 1]. Conversely, on topics involving the negative global impacts of biomass sourcing, storyline 1 plays down the scales involved. Drax [59] refer to the tiny percentage, *“45% of 1% of the total*” of global wood demand represented by biomass energy. The same holds in Europe, where *“the share of wood harvested in EU forests used in the energy sector has been rather stable”* [58 p 1]. When discussing European sourcing versus imports, emphasis is placed on the ability of European forests to meet demand, *“95% of the biomass consumed today is locally sourced in the EU”* [58 p 1].

#### Sustainability and forest management

Alongside carbon neutrality, sustainability plays a major part in this storyline, particularly in establishing an important role for biomass energy within the sustainable forest management (SFM) concept [56]. As the title suggests, forestry lies at the storyline’s core, and many of the actors involved in its telling are based in or strongly connected to the forest sector. *“Forest is continuously storing carbon [sic]*. *One aspect of a sustainable forest is to avoid the over-exploitation [sic]*. *It means that a stable wood stock should be permanently ensured in the forest*. *Such forest is a carbon neutral forest [sic]”* [60 p 1].

In recent decades European forests have been growing in terms of both volume and extent, growing stocks have increased by around 403 million m^3^ yr^-1^ during this period [61]. As Table 1 shows, this fact is variously adopted by storylines 1 and 2, each applying different frames to emphasise what this fact means from an economic and/or climate perspective. In this case, the surplus invites use. Expanding volumes are presented as a resource to utilise by intensifying existing forest management practices in Europe [57], and worldwide [49, 62]. *“A considerable part of this [increasing forest volume] could be harvested without causing any detriment to the sustainability of forest ecosystems”* [56 p 5]. The storyline also refers to a higher rate of C sequestration in younger versus older forest stands, therefore arguing that forestry in a business as usual or intensified management scenario will lead to high atmospheric carbon removal [49, 57] and greater climate mitigation benefit than maintaining older trees in the landscape [52].

Within storyline 1, biomass is presented as an opportunity, and the additional value biomass has gained via bioenergy demand provides the economic drive necessary to achieve *“better forest management*…*essential to maintain the health of the forest resource*” [49 film]. Reading between the lines reveals that texts variously define forest health as increasing volume and increment [63], or resilience to external factors such as pathogens or forest fires [57]. Not only does forest ‘health’ benefit, storyline 1 makes direct links between forest protection and biomass utilisation, as the resultant increase in economic viability of forest operations discourages owners selling or converting their forest for other land-uses [49, 51]. Economic incentives for management has led to a situation where “*demand for wood has over recent decades been the main reason why the quantity of wood those forests hold has about doubled”* [63]. Local communities benefit too: *“we have a new industry to help us now”* says an interviewee worker at Drax’s Mississippi pellet mill [49]. So, increased biomass utilisation for energy produces expanding, healthier forests, with local communities thriving. The benefits also extend to biodiversity [57]. In *The biomass sustainability story* [49], plantation based systems are presented as benign and natural. Cutaways of buzzing insects represent biodiversity, while replantation of millions of clear-cut hectares is presented excitingly as *“regeneration”*. These subtle indications are backed up by an interview later in the film, in which a local forester explains the benefits of clear-cutting systems for biodiversity and expresses his dismay that environmentalists question the stewardship role which foresters play.

#### The forest as a resource

The final assumption storyline 1 relies upon in its presentation of forest biomass as saviour is a technocratic definition of biomass as a resource. Forest biomass is presented as one of many forest products, a previously wasted material that is now mobilised [45]. *Sustainable forestry enables the bioeconomy*, states EUSTAFOR [64]. This new role for biomass fits within the emerging bioeconomy concept [65] and creates a strongly utilitarian rationale towards its utilisation. Within this storyline, forest biomass should be mobilised as far as possible and regulated primarily by market forces. As discussions around the details of RED II have developed, AEBIOM and others have been vocal in their lobbying to advocate that no restrictions are placed on the kinds of forest biomass that can be used, on the capacity of bioenergy facilities, and to resist the inclusion of the cascading use principle [66]. Such restrictions would *“hinder resource efficiency and innovation*, *creating excessive red tape and impacting negatively on the market”* [67].

Storyline 1 presents forest biomass utilisation as an essential part of decarbonising the European energy system, and its delivery an integral part of forestry practice. Therefore, renewable energy and forest management legislation should be naturally coupled. Related to this view is an interpretation that forest biomass utilisation is threatened by accusations from alternative storylines, with *“systematic bioenergy bashing”* falsely undermining its potential use as an essential renewable energy source, and the ability of forestry to deliver it [68 p 1]. On several occasions, actors such as AEBIOM and IEA Bioenergy have quickly responded to publications expressing more critical views. Such criticism is unwarranted, discrediting *“the honest work of hundreds of thousands of foresters and project developers”* [69]. Forest workers have been *“been doing it for years*” and are hurt by allegations that challenge their assumed stewardship role [49 film]. At the same time, the Brussels based AEBIOM organisation in particular aims to align its views with the political process around RED II. Following a vote in the European Parliaments environment committee in October 2017 in which it was decided that a risk based approach would be used to ensure sustainable biomass sourcing, AEBIOM applauded this as a *“pragmatic”* approach which takes *“reality on the ground”* (as described by storyline 1) seriously in the policy process [58 p 1]. The same outcome was widely criticised by environmental NGOs as unworkable and vague [70]. Storyline 1 proponents advocate for simplicity in the legislation, arguing that voluntary SFM practices already ensure sustainable sourcing, and any further regulation will hinder and unduly burden foresters in delivering the undeniable benefits of forest biomass [64].

In conclusion the forestry prioritised storyline 1 maintains that market governance and sustainable forest management guidelines ensure a sustainable supply of biomass in volume terms. As forest biomass is assumed carbon neutral, this will directly benefit climate change mitigation efforts by replacing fossil fuels, while delivering multiple co-benefits to forests, society and environment.

### Storyline 2: Climate focussed

As the title suggests, storyline 2 uses climate change mitigation as a lens for comprehending the forest biomass issue. Bioenergy should play a role within this, but this demands a nuanced, holistic analysis to ensure benefits are delivered and risks of potentially serious harm to environment, society and in particular to climate, are avoided. Matthews, Hogan [11 p 4] neatly summarise this climate focus, *“unless appropriate policy measures are taken to support sustainable bioenergy supply (in terms of impacts on GHG emissions)*, *particularly in the case of forest bioenergy supply*, *a significant increase in bioenergy use in the EU is likely to lead to a net increase*, *rather than decrease*, *in GHG emissions being contributed from bioenergy sources”*.

Two strategies are advocated by the storyline to reduce the likelihood of this potential net GHG increase. These are often presented simultaneously in the texts and not necessarily as mutually exclusive but are presented here sequentially for clarity. The first sees a diminished role for bioenergy in any form. In its place alternative renewable sources such as solar and wind would be prioritised, along with efficiency improvements and reductions in energy consumption [25]. The relative ease with which biomass fits into existing energy systems under RED, and RED II, is drawing investments away from these more climate beneficial energy sources [71], for example by extending the lifespan of coal power stations via financial support for co-firing or conversion to biomass only facilities [72].

The second pathway advocated by storyline 2 is a much tighter regulation of forest biomass sourcing and usage by RED II [11, 73]. The emphasis on these two strategies of avoidance or greater regulatory control differs depending on the level of criticism displayed by each text. Linking all strategies proposed by storyline 2 are calls for the regulation of forest biomass utilisation to be grounded in a comprehensive assessment of GHG and other environmental and social impacts. This includes the entirety of the forest biomass supply chain, as well as external influences on environmental carbon storage via indirect land use change (ILUC). The language reflects the audience. More academic texts such as those published by the UK’s Forest Research [11, 74], or detailed policy proposals such as WWF’s [71] *EU Bioenergy Policy*, outline life cycle assessment (LCA) approaches in which forest biomass utilisation would only be permissible where robustly monitored criteria were met. These must show mitigation benefits relative to counterfactuals considering alternative scenarios, such as if forest biomass was not utilised or regulated differently [11]. In the majority of the assessed texts however, more emphasis is drawn to the potential risks posed by forest biomass and how to avoid these, rather than technical descriptions of risk assessment procedures. This likely relates to their more generalist target audience (policy makers with little time, the lay public, etc.).

#### Carbon balance and scales

Assessing the impact of biomass harvesting upon forest and atmospheric carbon stocks requires attention to the timescale considered and the type of biomass removed. Biomass removal comes with a risk of reducing carbon stored in trees and soils even if forests are managed in sustainable yield terms, *“the net effect of harvesting on GHG emissions depends critically on how the harvested timber is utilised”*, and must be *“considered on a case-by-case basis together with the related payback period”* [75 pp 21–22]. While storyline 1 holds that all biomass forms are equally open to utilisation and market forces should govern end use, storyline 2 disagrees, stating that forest biomass sourcing must be carefully controlled to ensure climate benefit. An important means of this legislative control is a restriction of forest biomass utilisation to specific grades of forest products such as forest wastes and residues [76]. An open letter signed by almost 800 scientists including several IPCC contributors to the European Parliament summarises this message, strongly rejecting *“deliberately cutting down trees to burn them for energy”* [77]. The RED II proposal at the time of writing will result in “*profound harm to the climate and forests worldwide”* due to its blanket inclusion of all forest biomass in meeting renewable energy targets [78].

This rejection of deliberate harvests and larger grades of forest biomass relates to the importance storyline 2 attributes to carbon cycle timescale and the carbon debt concept, introduced previously. To use LCA terms, this position’s counterfactual is that if living trees remained unharvested these would go on sequestering or storing carbon for several decades at least. Larger grades of wood can also be used for longer lived products again storing carbon (such competition is discussed further below). Only wood wastes and smaller residues that would decompose rapidly or be burned in the forest, therefore emitting their stored carbon rapidly, can provide climate mitigation benefit if used to replace fossil fuel energy [77, 79]. *“Such wastes and residues [should] only benefit from subsidies or incentives if they have no significant alternative uses*…*(the cascading use principle)”* [71 p 2].

Another strand of this narrative of competition is that achieving the climate change mitigation targets set out in the Paris Agreement will require substantial additional carbon sequestration. This is most easily achieved through land management such as *“reforestation and forest restoration”* [71 p 3], as *“the only major global carbon sink over which humans can exercise some direct control is that due to land-based vegetation and soil”* [11 p 15]. Maintaining a major reliance upon forest biomass within the European renewable energy mix directly undermines this aim [80]. Greater emphasis is placed on the high carbon stocks in older stands, challenging storyline 1’s assertion [71, 75]. This view, that forest carbon stocks should be prioritised over energy consumption is again developed more strongly in storyline 3. These considerations of forest management’s importance for climate change mitigation calls into question the governance role provided by SFM guidelines, as advocated in storyline 1. Fern [80] point to the wide variation in management practices across Europe under the SFM umbrella. The voluntary nature of SFM and a lack of robust legal requirements regarding its application means that while some sourcing practices may be sustainable in terms of climate, environment and society, this is far from guaranteed. Storyline 2 therefore rejects [71] or advocates caution in the reliance upon SFM as a mechanism for ensuring a sustainable use of forest biomass [75].

#### Indirect effects

As discussed previously, the analysis revealed a dualistic use of scale as a rhetorical device by storyline 1, whereby the importance of forest biomass is emphasised by links to statistics pointing to the high percentage of renewable energy targets met by *“the most popular source of energy used in Europe”* [57 p 1]. Conversely when discussing the environmental impacts of forest biomass, storyline 1 downplays the scale issue. Storyline 2 presents an essentially inverted version of these arguments, with the risks emphasised by drawing attention to the magnitude and impact of large-scale operations, and a narrative that achieving sustainable consumption levels will require a significant reduction in forest biomass utilisation via regulation.

Europe’s heavy reliance upon forest biomass is presented as an unfortunate reality, an artefact of weak regulatory frameworks, which must be urgently addressed because the current and projected scale of consumption exceeds reasonable ecological limits, exacerbating negative environmental impacts. High demand for forest biomass is a direct result of its inclusion towards RED targets, following which *“the use of wood for energy increased by 75 million cubic metres”* while *“the use of wood that comes directly from the forest has grown more than the use of forest industry residues (24 versus 10 per cent)* [81 p 5].

Following on from this argument is that if holistic sustainability considerations in terms of climate, environment and society were robustly regulated, the amount of available forest biomass would reduce significantly [25, 71], and could not sustainably meet European scale renewable energy targets in their current form [76]. Fern [82 p 5] summarise their stringent criteria for acceptable forest biomass, stating, *“biomass must be additional to be ‘carbon neutral’”*. In such a case any biomass must not come from increased harvesting, but instead be sourced from harvest- and forest product industry residues that would have rapidly decayed or been disposed of without carbon storage benefit.

Further opposing framing of concepts was found in storyline 2’s interpretation of the ongoing expansion of European forests, used as rationale for intensified harvesting rates by storyline 1. A letter to the Guardian from several senior climate scientists presents this as forests recovering from over-harvest, evidence that forest biomass cannot sustainably deliver energy at scale: *“By 1850*, *the use of wood for bioenergy helped drive the near deforestation of western Europe even at a time when Europeans consumed relatively little energy*. *Although coal helped to save the forests of Europe*, *the solution is not to go back to burning forests”* [78]. Current developments in European energy regulation are likely to return us to this point of overconsumption, as *“more than 100% of Europe’s annual harvest of wood would be needed to supply just one third of the expanded renewable energy directive”* [77].

The storyline advocates several means for addressing the scale issue, such as enforcements on efficiency ratings, or requiring co-generation of heat and power for plants over a certain capacity threshold [71, 83]. Fundamental though is a stated or implied stance that if the many concerns raised were taken into account in a holistic assessment of sustainability, many currently mobilised forest bioenergy streams would not meet these standards. Instead, forest biomass should be utilised on a smaller scale in specific situations where it is beneficial or unavoidable, but achieving this would require fundamental changes to the renewable energy legislation.

The more critical storyline 2 (and 3) texts frequently use variations of *“Burning trees whole in our power stations”* [84] as a rhetorical device to evoke an emotional response in the reader—the tragedy of a wilderness burned to feed our insatiable demand for energy. Storyline 2 points to cases and statistics to support this [81], in contrast with storyline 1 which holds that specific harvests for bioenergy do not happen but rather market demand guides end use following routine forestry operations. The film *Bioenergy*: *the ugly truth* [85] presents several such worst-case examples of where economic incentives drive harvest intensification, negatively impacting climate, biodiversity and local communities. These case study examples are tied to recommendations that bioenergy policy *“must not cause direct or indirect destruction or degradation of forests or other ecosystems”*, particularly those *“with high biodiversity and/or carbon storage value”* [25 p 7]. As seen below, the more critical stance of storyline 3 refers to cases of environmental destruction as evidence that forest biomass will never be the benign, renewable energy source as claimed by its supporters.

### Storyline 3: Critical

The third and final storyline paints a highly critical picture of forest biomass energy, emphasising major risks to climate change mitigation efforts, environment and society, and rejecting proposals that sustainability criteria or other governance mechanisms will be adequate to reduce these risks. At the core of this storyline lies a rejection of bioenergy overall from inclusion in the EU RED, as seen in a 2015 open letter from 120 civil society organisations including the UK NGO Biofuelwatch and the Danish national FoE chapter NOAH [86]. The call was reiterated in response to a January 2018 European Parliament RED II vote [87].

#### Rejection of bioenergy

It is this call for bioenergy’s removal from RED legislation which differentiates storylines 2 and 3. The narratives of destruction, risk and avoidance of forest biomass are present in both, and indeed are often expanded upon in greater detail with greater reference to scientific literature in storyline 2 texts. The NGOs Fern, BirdLife and Transport & Environment for example have shown consistently critical narratives in storyline 2, releasing several reports emphasizing the high risks of the EU’s ongoing reliance upon forest biomass yet concluding with calls for improved governance and sustainability standards within existing regulatory frameworks [70, 80, 85].

However as the finalisation of RED II was approached, the line between these two storylines became less clear. For example following a June 2018 trilogue session on RED II between the European Parliament and Commission, Fern [81, 88] responded with press releases and a report using language again aligned with the critical storyline as in their earlier publications. However, they made no policy recommendations, not directly calling for a rejection of forest biomass from EU renewable energy targets, but strongly implying this as the only sensible option given the magnitude of the associated risks. The rejection of bioenergy and forest biomass from the RED is supported by narratives linking current and projected bioenergy trends with the exacerbation of climate change, a crossing of several ecological limits, and various negative societal impacts including human health and global social justice. The content of these narratives will be summarised in turn below.

#### Negative ecological impacts of forest biomass

Storyline 3 discusses multiple negative impacts of forest biomass upon climate change mitigation efforts; the physical GHG emissions during combustion and following direct land use change, the *“perverse outcomes”* where policies favouring bioenergy reduce investments in alternative low carbon technologies and societal transitions, and indirect emissions via leakage in consumption of land and resources [86]. The carbon debt concept plays a strong role here, presented as an unavoidable and damning oversight in the EU’s forest biomass energy usage, rather than as grounds for LCA approaches to optimise the carbon cycling timescale as advocated in storyline 2. Instead, existing *“flawed methods for calculating [GHG] emissions”* and an absence of proposals *“for any credible*, *independent verification and auditing system”*, negates claims that forest biomass presents a low carbon alternative to fossil fuels. If *“all direct and indirect [climate] impacts are accounted for”* forest bioenergy would *“increase rather than decrease carbon emissions when compared to fossil fuels”* and therefore cease to appear as a viable solution in EU renewable energy targets or climate change mitigation obligations [24 pp 1–5]. Beyond climate change, the storyline draws attention to the unavoidably destructive nature of industrial scale bioenergy from an ecological point of view. With several ecological limits crossed this calls into question the very definition of forest biomass as a renewable energy source, defined by the IEA as resources *“replenished at a faster rate than they are consumed"* [89 p 2]. While storyline 1 states that SFM practices ensure a steady timber supply and that forest biomass utilisation is therefore renewable, storyline 3 presents the resources under discussion (and management) as more than the trees in the forest.

Firstly the storyline questions the fundamental assumption that forest biomass itself is a renewable resource, *“there is no guarantee that all biomass that is burned is replenished*, *and it is never replenished ‘at a faster rate’ than it is consumed”* [86]. A second over-exploited resource is the ‘natural’ status of forest and other ecosystems, as forest biomass entails greater *“clearcutting of forests and*… *destructive logging practices*, *while*… *plantations for energy replace forests*, *biodiverse grasslands and other ecosystems”* [89 p 2]. The narrative paints natural forests as threatened by *“industrial logging”* and replacement with *“monoculture tree plantations”* [86]. Further resources include *“soils and freshwater*…*”* which *“are effectively ‘mined’”* [89 p 5]. Air quality too is threatened by the use of *“agrotoxins”* in plantations, plus the production and combustion of pellets [24]. Finally, the land itself is a resource under threat, with *“wood-based bioenergy*…*driving a large share of land-grabs”* [89 pp 2–3]. As each of these resources is depleted beyond acceptable limits (if these exist within this narrative) by forest biomass consumption, its renewable status is fundamentally rejected.

#### Negative societal and health impacts

Alongside these ecological arguments, storyline 3 presents several negative societal impacts from forest biomass, particularly in relation to social justice issues. In this narrative, Europe’s growing demand for biomass will lead to *“landgrabbing*, *displacement and other injustices”* with globalisation of supply chains leading to particular threat to *“the livelihoods of workers*, *farmers*, *Indigenous Peoples and other communities*, *particularly in the global South”*. There are also feminist narratives present, again with a focus on the ‘global south’. Lost land and conversion to monocultures exert *“particularly serious impacts on women”*, leading to *“increased work load*… *and also in an increase in violence against women”* [86].

Human health is also threatened by the air pollution associated with combusting biomass, which produces *“particulates*, *nitrogen dioxide*, *dioxins and furans and heavy metals that impact public health and reduce quality of life as well as life expectancy”* [89 p 3]. The health issue is another point where the boundary between storylines 2 & 3 has recently blurred. In 2018 Fern released a detailed scientific report on the impacts of biomass upon mortality rates, respiratory diseases, and the related economic impacts this entails upon healthcare, lost productivity and the value of human life. The report concludes “*an increase in use of biomass combustion to meet [renewable energy targets] would have a significant impact on human health”* [90 p 42]. As discussed above, in preceding years Fern’s outputs though critical in their portrayal of forest biomass’s impacts, can generally be linked to wider narratives in storyline 2 calling for risk mitigation via improved renewable policies within existing EU regulatory systems. Notable here then is the critical tone of the reports final conclusions, which stress that existing wording in the proposed RED II is inadequate to address air quality and associated health impacts, and finally that *“other renewable technologies*… *may offer a solution with substantially lower external costs”* [90 p 44].

#### Regulation

On regulation, storyline 3 differentiates between small and large-scale bioenergy in its narrative. Providing the impacts outlined above can be avoided, *“small-scale local bioenergy schemes could still attract support*, *for example under Rural Development programmes*. *In fact*, *community-based bioenergy schemes often benefit from this type of support already*, *rather than from the subsidies that stem from the Renewable Energy Directive*, *which disproportionately boost large-scale industrial schemes”* [86 p 2]. Under existing rules and the proposals for RED II however, *“proposed biomass sustainability standards are so weak as to be effectively meaningless”* [24 p 4], while at the same time EU subsidies have encouraged an increase *“in the burning of wood*…*in domestic appliances as well as industrial facilities*. *The latter includes large power stations*, *some of which were originally built for coal combustion*, *and smaller facilities specifically designed to burn wood”* [90 p 6]. The storyline portrays a lack of trust in existing regulatory procedures, under which *“standards and certification schemes are applied only to specific loads of biomass”*, but that the existing *“self-regulation by companies and their chosen consultants”* will enable companies to avoid accurately reporting their impacts. *“As the Volkswagen scandal has shown*, *standards and even regulations are ineffective without strict independent enforcement”* [86]. This combination of weak regulatory frameworks vulnerable to manipulation and at the same time encouraging large-scale biomass utilisation (and the climate, environment and societal impacts this has), lies at the core of storyline 3’s rejection of biomass from the proposed EU RED II. Forest biomass is variously presented as a false solution, competing with *“wind and solar power and a distraction from the urgent need to reduce the EU’s wasteful energy use”* [24 p 2]. Its inclusion within Europe’s bioeconomy intiatives is deeply problematic here, as *“the false belief in a future ‘bioeconomy’ is further delaying urgently needed measures to drastically reduce the EU’s energy and resource use*” [89 p 3]. Bioenergy policy instead *“needs to be part of much more far reaching policy changes to reduce the EU’s excessive and wasteful use of energy and resources and to move away from the current growth-oriented economic model”* [89 p 6].

The three storylines presented above show that Hajer’s [26] storyline concept provided a useful tool for conceptualising the forest biomass debate, identifying narratives which together form consistently structured discourses (storylines) emerging from the gathered texts. Table 1 summarises where these storylines differ regarding their treatment of several important narrative elements. The following section will look more closely at how sustainability and nature are presented in the three storylines above, and the roles, which expert knowledge and science play as the involved actors compete to assert a position of truth regarding their interpretation of the forest biomass issue.

### Explaining differences between the storylines

The term sustainability is perhaps the best example of where ambiguous definitions of widely used terms leads to misunderstanding, and potentially to selective misrepresentation of facts. This is apparent in the forest biomass case, where different interpretations and applications of sustainability within each storyline provide an explanation for ongoing disagreement around the question: what exactly is sustainable forest biomass? Each storyline states a claim towards sustainable forest biomass usage, yet none of the analysed texts offer a comprehensive definition of sustainability. This is not surprising, as the storyline concept enables actors to mobilise around particular ideas without continually redefining what is meant by key terms [26] as each actor or organisation presumably has a good understanding of what sustainability means already, for them. Yet in this environment vs. environment discourse [16] where each actor uses similar terminology to refer to entirely different assumptions, misunderstanding and frustration emerge, although one storyline 2 text recognised that *“an ecologist*, *forester and social scientist would all view the sustainability of the same forest through different lenses”* [80 p 6]. Below we go through how each storyline consider sustainability and related concepts.

#### Sustainability in Storyline 1

**Fig 2:**
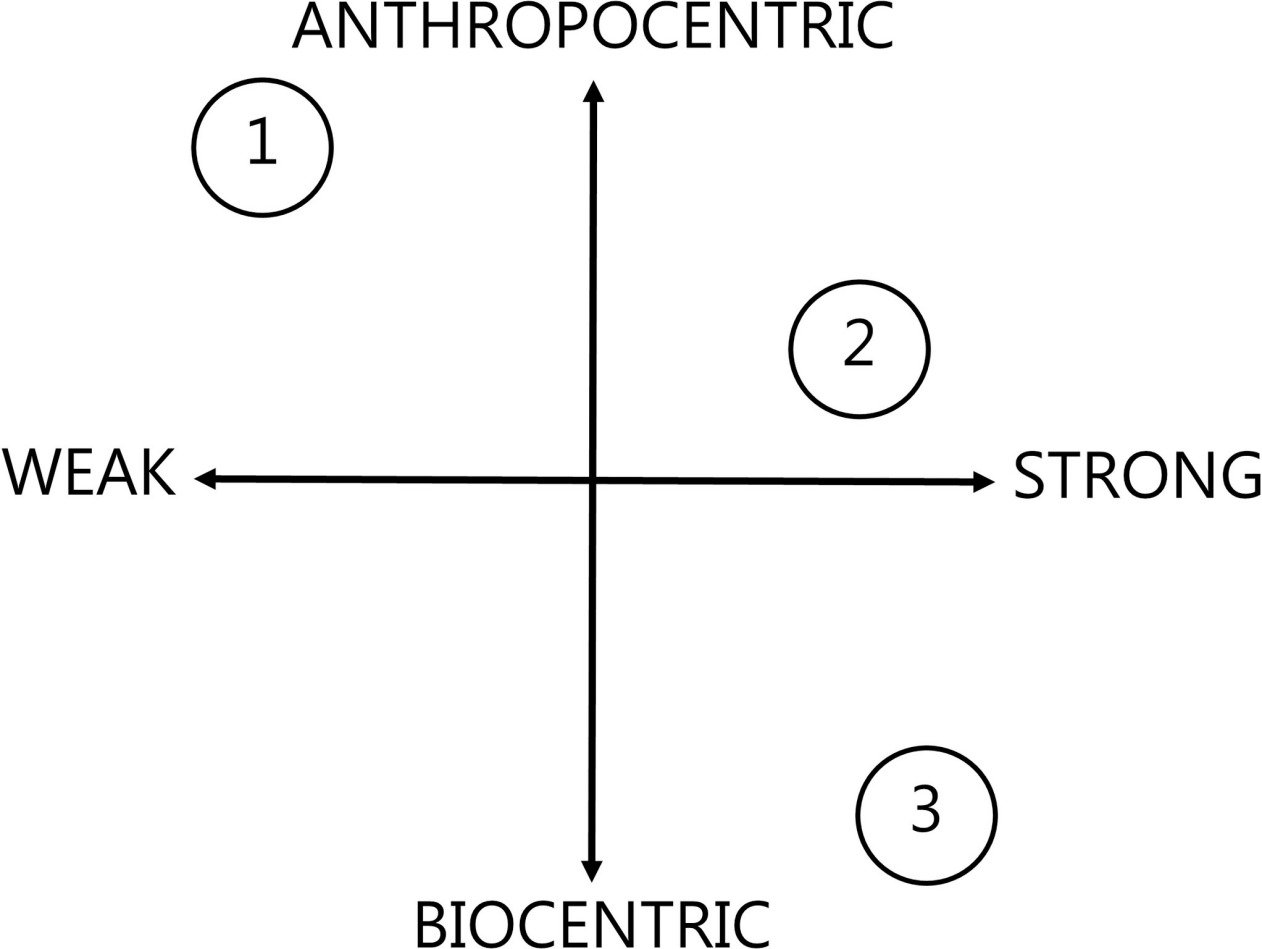
The positioning of storylines 1, 2 and 3 in terms of their definitions of sustainability and nature in relation to the forest biomass issue. Sustainability is presented on the horizontal axis as a spectrum between strong and weak interpretations of sustainability, after [32]. As explained within the text, this is interrelated with storyline definitions of nature, presented on the vertical axis here as a spectrum between an anthropogenic or biocentric prioritisation.

As seen, biomass advocates point to SFM as ensuring sustainable yields and therefore sustainable biomass. Their sustainability is focussed on the ability of forests to be continually managed for biomass purposes:*”for [forest biomass] to be truly beneficial and sustainable we need to*…*meet the demand of today*…*and tomorrow”* [57 p 4]. Thus the forestry prioritised storyline 1 is positioned with a firmly economic interpretation of sustainability, corroborating Söderberg and Eckerberg’s [16] findings that forestry sector actors prioritised ‘green growth’ to a large extent in their framing of the forest biomass issue. Within this storyline a more or less business as usual approach to governance is advocated, with market regulations and sustainability criteria presented as sufficient to sustain a consistent, safe supply of forest biomass for all end uses, and with bioenergy providing a new window of opportunity to support the forestry sector. Returning to the concepts of weak and strong sustainability [32] finds storyline 1 aligning with the weaker end of the spectrum, as shown in Fig 2.

Bioenergy presents both an opportunity for forest managers in economic terms, but also provides a new platform for forestry to continue its supportive role under the name of progress, particularly given the inclusion of forest management within Europe’s drive towards establishing a ‘bioeconomy’ [64, 65]. This is reflected by several storyline 1 texts, for example AEBIOM [48 p 1] describe a forestry sector *“working tirelessly to accelerate the energy transition*… *Limiting the development of Europe’s largest source of renewable energy is a dangerous game*, *as in many cases bioenergy represents the best option to lead the energy transition”*. De Gemeynt and MSG Sustainable Strategies [43] also identifies the bioeconomy and “Renewable raw materials” as an independent perspective, while we have identified this theme as part of storyline 1. Relating this viewpoint back to the weak sustainability concept finds that storyline 1 interprets managed forest ecosystems as cultivated natural capital [33], grown for anthropogenic ends, rather than natural entities with inherent value. The interaction between the storyline’s definition of sustainability and nature is demonstrated in Fig 2. Rather than the biocentric ethical appreciation of forests as entities in themselves, the argument here is that management can bring forests closer to an ideal form, even more so with greater demand for biomass energy [62 p 2].

#### Sustainability in Storyline 2

With storyline 2’s prioritisation of climate change mitigation and the proliferation of environmental NGO actors among its proponents, it is not surprising that sustainability is presented in much broader terms here, environmental, societal and economic goals are considered interdependent, although a prioritisation of the first two is often apparent within the texts. There is a strong view of sustainability, with forest ecosystem functions for biodiversity and particularly climate change mitigation viewed as critical natural capital, and therefore irreplaceable by manufactured alternatives [33, 91]. That said, the storyline’s presentation of how forests as natural ecosystems relate with anthropogenic systems is less strongly defined than the more polarised storylines 1 and 3. Fig 2 therefore shows storyline 2 located at a midpoint on the anthropogenic-biocentric spectrum regarding this definition of nature, although this positioning varied between texts depending on their form and target audience. Forest destruction is presented as an inherent moral bad in texts taking a more emotive tone [77, 85], while other more technical reports make stronger references to the role forests play towards societal climate change mitigation efforts [11, 75]. Storyline 2’s emphasis on LCA approaches to understand and contain the risks presented by forest biomass [11, 71, 74] means that elements of nature necessarily become monetised within the ecosystem services concept [32] particularly in texts directly referring to EU bioenergy policy. As discussed in the storyline 2 description, along with a broad definition of sustainability is a rejection of the notion that forest biomass is inherently sustainable even under SFM, with *“tensions between some of the objectives of SFM—especially between demands for increased extraction of biomass from forests and the contributions made by the same biomass in situ to soil fertility*, *biodiversity and protective functions*. *Other synergies and trade-offs exist in the way in which forests’ interaction with climate change mitigation is managed”* [75 pp 2–3]. These apparent contradictions between a default assumption of sustainability under SFM rules and the additional sustainability objectives prioritised by storyline 2 result in calls for a far more restrictive legislation of forest biomass, such as a restriction to wastes and residues, and a rejection of *“burning whole trees”*, or *“deliberately harvested wood”* [78].

#### Sustainability in Storyline 3

This narrative of constraint is most visible in the critical storyline 3, in which the multiple ecological limits crossed by large scale industrial utilisation means that beyond restricting sourcing, EU renewable energy policy should reject forest biomass entirely along with bioenergy in general. This view contains a paradox in that rejections of bioenergy within the modernist technocratic discourse [31] are usually accompanied by endorsement or allowances for small scale bioenergy in connection with these actors’ simultaneous discussion of global social justice issues and feminism [89]. However, this narrative opens up further unanswerable questions. As Sandilands [92] explains the concept of natural limits commonly present in environmental discourses are just that: concepts, socially constructed. Therefore, any attempt to precisely define what a minimum threshold or capacity bioenergy system can be is not readily answerable by ecological science and remains a point of contention in environmental circles. This stems from a feature of radical environmentalism in general as grounded in a biocentric resistance to the problems produced by technological and scientific advances, but then requiring the same fields to define, understand and solve these problems [29, 31, 93]. This generates a tension between *“acceptable normative understandings of natural limits for resource use”*, and a *“functionally defined*, *systematic”* rationale for resource management [28 p 100] which the environmentalist response to bioenergy must navigate. Having said that, gaining insights into how differing prioritisations influence the acceptability of forest biomass utilisation among environmental actors can inform policy and consultation approaches. With governance attempts ignoring social and ethical ideas likely to fail [94] and ethical questions often beyond measurement by conventional scientific approaches [29], it is clear that coming to reasonable consensus on the forest biomass issue will require a far broader approach than is arguably being adopted currently.

Relating the storyline 3 vision of sustainability within the strong versus weak categories [32] finds an alignment clearly with the former as seen in Fig 2. These more radical environmental groups generally hold a deep scepticism of sustainable development and bioeconomy concepts and the global environmental governance institutions behind them [31, 89]. These actors’ strongly biocentric discourse rejects terms such as ‘natural resources’ and certainly ‘natural capital’, due to their ethical prioritisation of ecosystems as essentially beyond valuation in monetary terms. Proponents of storyline 3 might also reject any characterisation of their discourse using these terms, which themselves emerged from institutions they may treat with scepticism [91]. Explaining the storyline 3 discourse using the sustainable development parlance however finds that the multiple ecological boundaries which forest biomass is said to cross relates easily with the critical natural capital concept [33]. This exact point is emphasized in De Gemeynt and MSG Sustainable Strategies [43] that identifies an individual perspective related to ecology and planetary boundaries. Forest bioenergy threatens to undermine human wellbeing in a number of dimensions, including health, land rights, clean water, healthy soils, and climate [24], each definable as critical and irreplaceable. The strongly biocentric ethos of the engaged actors here means that ‘capital’ also takes on a wider relevance beyond human monetary value to encompass the roles provided by forests as ecosystems with no equivalence to the capital gained through technological development and growth.

As seen, storyline 3 holds nature as an entity in itself, with *“people [and]…forests”* threatened by forest biomass utilisation [86 p 2]. This view then puts forests as an entity alongside humans, with rights which must be considered and prioritised before monetary economic gain and ultimately requiring a move *“away from the current growth-oriented economic model”* [24 p 6].

### Interactions between the storylines

The analysis found the use of knowledge as a political tool, with actors seeking to assert the credibility of their arguments, to align these with policy making discourses, and to discredit the arguments of other actors by presenting them as unscientific. Considering these uses of science also revealed differences between the storylines regarding the prioritisation of specific knowledge types.

A variety of documents were used to support statements made in the texts, from peer reviewed science, official statistics, grey literature consisting of reports produced by NGOs and companies, or by commissioned thinktanks and research groups. The analysis found that while each storyline made similar use of citations and references, this depended on the text context and target audience: reports presenting a more technical appraisal of forest bioenergy included more reference to technical reports and peer reviewed literature as would be expected. A major narrative element of storyline 1 is an assertion that the storyline represents *the* pragmatic approach, grounded in forest and economic science, and making policy recommendations based on facts on the ground. Thus, storyline 1’s proponents assert that they are the experts while alternative narratives are based on flawed science at best, and strongly prioritise arguments supported by official statistical evidence and industry reports [58], suggesting a lesser prioritisation of qualitative approaches considering aspects such as society and ethics. From this self-proclaimed position as expert comes allegations of attempts to mislead in the other storylines, often with reference to specific texts located at the more critical end of the spectrum. These exchanges reveal an underlying difference in the conception of risk and the precautionary principle between actors generating each storyline. AEBIOM’s response to the film *Bioenergy*: *the ugly truth* [85] is a good example of this. The BirdLife film presents several case studies as evidence of the environmental and climate damage caused by perverse incentives for bioenergy in European legislation. AEBIOM [69] attack the *“risky game”* played by the film’s creators, who are *“seeking to tarnish the reputation of bioenergy*, *a central solution to providing clean energy”*. *“It would have been more constructive”* AEBIOM argue, *“to comprehensively report on the great number of sustainable examples of bioenergy projects spread across Europe*. *It would be a more credible*, *but far less sensational narrative”*.

The exchange shows that BirdLife and Transport & Environment [85 film] view case examples of negative bioenergy impacts as evidence that the current legislation enables a *“paradox”* situation in which *“tax payers are subsidising the destruction of forests”*. AEBIOM on the other hand do not follow the precautionary principle here, rather arguing that while some *“poor practices need to be addressed”* critics should understand that biomass is *“here to stay”* and further dialogue should proceed on *“the basis of non-fictional grounds”*. Rather than environmental damage, the risk that AEBIOM prioritise is that if critics succeed in *“tarnishing”* the reputation of biomass, *“we are left with only one alternative*: *an increased use of fossil fuels*, *resulting in higher greenhouse gas emissions”*. In this approach, the proponents of storyline 1 use their position as forestry experts to challenge NGO positions as based on flawed science.

The analysis found numerous examples of specific instances where there has been a rapid response to reports and media output criticising forest biomass, in which pro-biomass actors have unpicked the arguments and used their scientific credentials to assert that any allegations of environmental risk are unwarranted. In 2017 for example, IEA Bioenergy responded to a critical report by UK think tank Chatham House [95], producing a rebuttal document which unpicked the inaccuracies in original text. IEA conclude that the report *“does not present an objective overview of the current state of scientific understanding with respect to the climate effects of bioenergy”* [51 p 2]. This message of science’s proper usage is strong throughout the IEA Bioenergy response as well as the argument which ensued. The four co-authors’ credentials as scientists and specialists in forest and energy industries are prominently displayed at the start of the text, ensuring the reader that this is the voice of qualified experts.

Storyline 2 similarly presents scientific expertise as a rhetorical device, but prioritising climate science in the place of forest and resource management expertise and also prominently displaying it to gain credibility [77, 95]. A further example of the differential application of scientific evidence is the treatment of the CO_2_ emitted from forest biomass versus fossil fuels. One of the key arguments from IEA Bioenergy against the Chatham House report was that it *“blurs this distinction between fossil and biogenic carbon*, *which is misleading”* [51 p 1]. The conceptual choice made by proponents of storyline 1 here is noteworthy. While CO_2_ has been normatively defined as pollution in wider climate change mitigation discourses, storyline 1 holds that while moving fossil carbon to the atmosphere results in ‘matter out of place’ to borrow Mary Douglas’s [96] classic phrase, the same molecule can be emitted with impunity providing it is named bioenergy. The persistence of this carbon neutrality assumption in storyline 1, although shown to be overly simplistic [46], can also be explained by its relationship with forest science. As Ter-Mikaelian, Colombo [17] explain, *“from a forest manager’s perspective*, *this logic [of carbon neutrality] can be appealing because it appears to fit a sustained yield paradigm”*. These arguments are also displayed in De Gemeynt and MSG Sustainable Strategies [43] that has climate as a point of disagreement between bioenergy proponents and opponents.

The time element between carbon emission and uptake is a fundamental consideration regarding the climate benefits of bioenergy versus fossil fuels [47], and as Table 1 shows time is variously applied across the storylines. Whether this results in a clear delineation between the CO_2_ produced by either source is essentially a framing device as the climate makes no such distinction, nor do the IPCC analyses [53] often cited by texts making this argument [51, 97]. Alongside time, spatial scale is another point of contention between the storylines, a further location where contradicting facts emerge between actors across the spectrum of scientific-ethical narrative prioritisation. In their study on the political ecology of biomass energy, Van der Horst and Evans [98] discuss scale, which along with nature and sustainability they argue can be considered a socially constructed concept often co-opted for political purposes. They find scale as dynamic within the forest biomass discourse, *“never set…perpetually disputed*, *redefined*, *reconstituted and restructured”* [98 p 177]. The resulting differential treatment of scale can be observed here, as seen in the opposing framing between storyline 1 and 2 regarding how forest biomass impacts relate to the scale at which it is applied. Storylines 3 goes further still, with the negative impacts of large scale bioenergy forming the fundamental argument against its inclusion in EU renewable energy policy as seen in the following passage from NOAH, Biofuelwatch [89 p 6]: *“Thus we see that the scale of industrial bioenergy is a problem in itself*. *This means that standards and certification cannot ensure sustainability because they apply only to specific loads of biomass or biofuel*, *and have no impact on scale and expansion*. *On the contrary*, *they may add to the problem by legitimising large-scale bioenergy use in the eyes of the public… Bioenergy can only be produced and used sustainably on a local and small-scale basis*. *This cannot be appropriately regulated under current EU legislation*, *but needs to be managed at local level*.*”* As described by Sandilands [92], boundaries and scales are difficult concepts in environmental issues due to the necessarily value laden prioritisations involved in choice making, which thwart a wholly scientific approach in their definition. Van der Horst and Evans [98] apply this thinking to biomass energy, finding that *“the ‘systems boundaries’ that scientists draw around their lifecycle analysis can and should be critically assessed as they present an explicit social framing*: *what alternative options can be explored for these materials*, *for the land on which they grow*, *or for the fuels which they are supposed to displace*?*”* [98 p 190]. Bentsen’s (2017) meta-analysis [12] lends support to this conclusion. He found wide variation across forest biomass carbon debt studies were primarily attributable to researcher choices. Thus, establishing which carbon cycle timescale is climate safe and at what magnitude forest biomass can be utilised can be indefinitely debated, and requires a broad and holistic consideration of science and societal values.

### Discourse coalitions

The analysed actors displayed a differential connection with their associated storylines. A cluster of companies, industrial organisations and engaged individuals can be consistently observed around the forestry prioritised storyline. For this group, the storyline concept can be seen to fulfil Hajer’s [26] definition as a means of shorthand to reduce the complexity of the forest biomass issue down into the specific components which matter, particularly its role in enabling good forest management, and the wood industries and rural livelihoods it supports. Over the decade investigated, the boundaries between storylines 2 and 3 became increasingly blurred, as observed previously regarding the human health issue, and seen in the tendency for environmental NGOs previously located in storyline 2 to display increasingly critical narratives. Our result, that stakeholders or storylines cannot always be distinguished is apparent from De Gemeynt and MSG Sustainable Strategies [43] that have 5 different “perspectives”, which are roughly the same as our storylines. Their classification shows overlap with some of our storylines, but they also analysed additional discourses, especially relating to biomass use in their national context.

Both WWF and Fern have hardened in their recent discourse. This is seen in a movement beyond criticisms of specific instances where forest biomass presents environmental and social risks but encouraging a legislative approach in containing these, towards reports which discuss mounting concerns in detail, often with emotive and apocalyptic overtones, but suggesting no political solution. As Hajer and Versteeg [99] point out, actors form coalitions around storylines with the often-false assumption of mutual understanding, as the process of simplification conceals underlying discursive complexities. The effects of this can be observed in this gradual transition of environmental NGOs toward more critical standpoints. Another possibility is that new information emerges which does not easily fit into the storyline previously associated with an organisation’s discourse. An example here is the inclusion of health impacts in the narrative output of Fern [90], a topic previously only discussed by the more critical NGOs in storyline 3 [24]. The arguments within this report are not easily reconciled with the likely legislative output from RED II given their much broader policy ramifications in terms of healthcare, air quality, and social mobility (as the report finds that poorer communities are likely to be impacted most strongly by forest biomass operations). Together, this means that this report’s findings clash with those previously published by Fern in which they seek to influence EU policy to contain the risks presented by forest biomass without deeply changing the system at large, and represents a potential move to a more critical position for the organisation as a whole.

### Navigating the forest biomass debate

**Fig 3:**
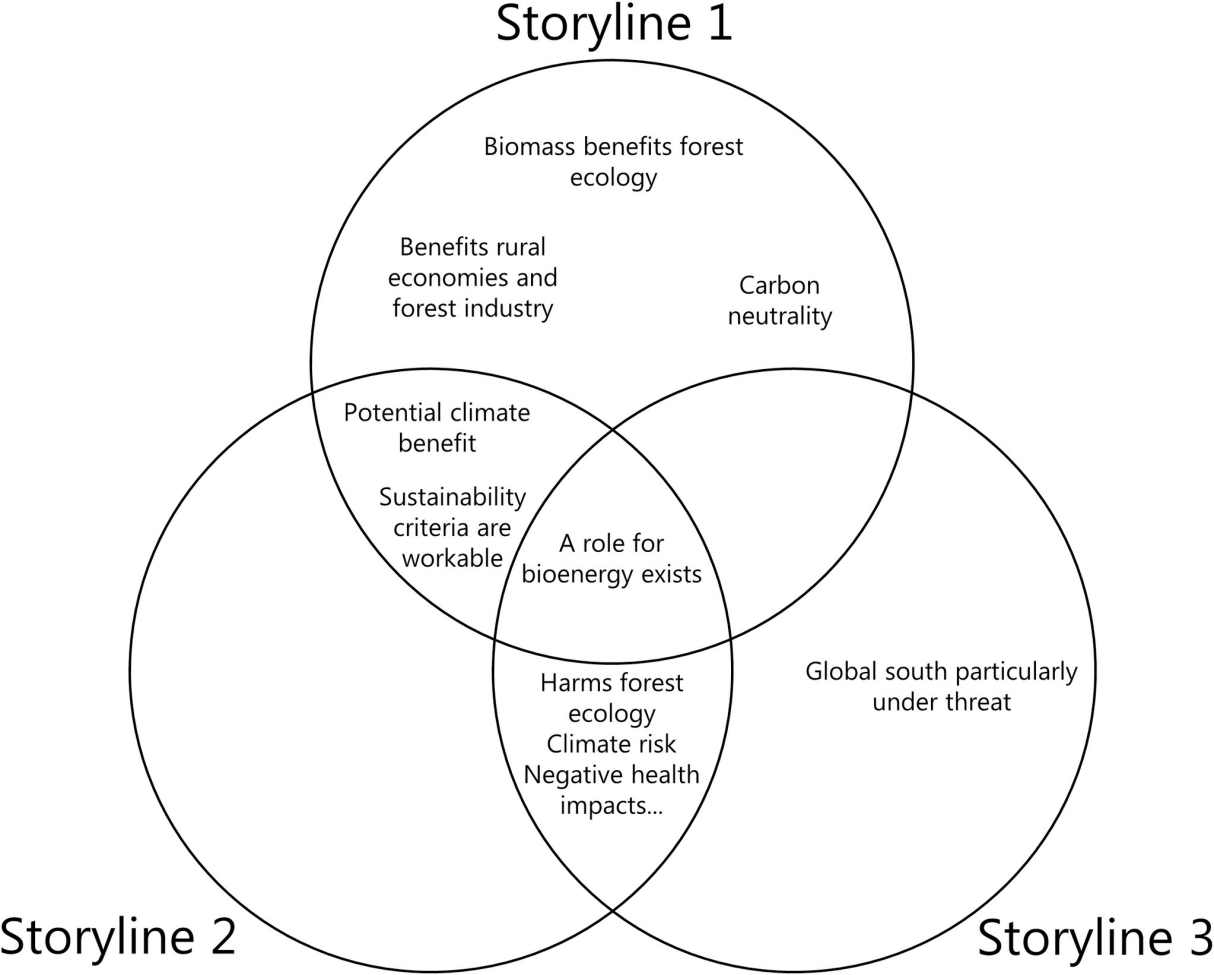
Venn diagram illustrating the points where the discourses presented within each storyline overlap or diverge. Each point relates to the benefits or risks of forest biomass energy as assumed by each storyline.

As seen, the three competing discourses/storylines outlined in this study contain multiple points where differing underlying values and definitions of key terms, particularly sustainability and its influence upon interpretations of nature lead to conflicting interpretations of the forest biomass issue. Clearly, each of these storylines hold truths for the actors engaged in their telling [100], which explains the contradictions present in the debate in which all sides assert a position of science based fact regarding the issue, and often discredit or ignore the positions of those in disagreement. That said, there are also multiple locations where despite their differences, the discourses converge. These points are displayed in Fig 3, which illustrates where specific narratives are repeated to some extent within two or more storylines, along with some non-overlapping narratives for reference.

The most striking observation from Fig 3 is that all storylines present a role for forest biomass in some form. In De Gemeynt and MSG Sustainable Strategies [43] it is noteworthy as well, that all of their “perspectives” see a role for biomass as well, thus agreeing with the results of our study. While our storylines 1 and 2 discuss the possibility that forest biomass could provide climate benefit (the former more strongly so), the critical storyline 3 comes with a rejection of bioenergy entirely from EU renewable policy. At the same time however, the storyline states that *“bioenergy can provide a sustainable energy option*, *but only when produced on a small-scale basis for local energy needs”* and *“could still attract support*, *for example under Rural Development programmes*. *In fact*, *community-based bioenergy schemes often benefit from this type of support already*, *rather than from…subsidies…which disproportionately boost large-scale industrial schemes”* [86 p 2]. This statement warrants further consideration as it reveals that as well as considering some aspects of bioenergy to be beneficial for climate and environment (as contained within the storyline’s definition of sustainability), the benefits can extend to rural communities via support for community energy ownership. AEBIOM [101 p 1] too express their view that *“communities should benefit”* from forest biomass, but go on to argue that the kinds of restrictions called for by storyline 2 and in particular 3 will undermine this, by adding *“additional burdens for thousands of small and medium size operators across Europe*, *while deterring the development of hundreds of local projects”*. These deeply held disagreements regarding the impacts of forest biomass are unlikely to be resolved easily but identifying points of overlap in the prioritisations within each storyline as seen regarding the potential benefits for communities here, could provide an opportunity for more constructive dialogue.

This follows the findings described by Upham, Riesch [10 p 514]. They concluded that given the huge diversity in knowledge(s), assumptions and values, actors engaged in the forest biomass debate conceptualise risk and uncertainty in a multitude of equally rational and valid ways. The challenge in gaining consensus stems from the polarised world views of the actors involved, and the discursive stability created by deeply held values [42, 44]. The relevance of forest biomass within the emergent bioeconomy paradigm or discourse is a good example of how divisive the issue will likely remain. As described by Pülzl, Kleinschmit [65] Europe’s focus on the bioeconomy has evolved from previous discourses on ecological modernisation and sustainable development, and follows their assumptions of technocratic assimilations of nature towards largely anthropogenic ends, with economic considerations greatly eclipsing those of society and ethics. Forest management’s inclusion within the European bioeconomy is roundly accepted in storyline 1 [64]. Storyline 3 counters these claims on forest biomass, which is not surprising given the deep scepticism of radical environmentalist circles to the hegemony of growth and technocratic environmental policy [31]. This is apparent in the texts criticism of ‘industrial’ forest management and in their calls for alternative societal pathways where growth is no longer prioritised, ultimately resulting in a far lower energy demand [89].

The critical storyline’s rejection of industrial biomass utilisation may appear naïve to the forester accustomed to the use of large machinery to reduce environmental damage and improve efficiency. In fact, this narrative element illustrates a fundamental ideological concept, in terms of the deep environmentalists rejection of growth as the sole indicator of progress, and the reliance upon scientific and technocratic manipulations of the natural world in maximising capital gain [31].

## Conclusion

The three storylines presented here represent three competing discourses regarding forest biomass usage in European renewable energy: forestry prioritised, climate focussed and critical. Each of these are promoted by actors aiming to gain discursive hegemony on the issue, both in terms of the impact of their discourse upon EU policy making and in the eyes of the public.

Hajer’s [26] storyline concept was highly relevant for conceptualising the forest biomass debate, explaining the presence of narratives persistently shared between the texts forming each storyline. For example, assumptions of carbon neutrality and the inherent sustainability of forest biomass dominated the forestry prioritised storyline, invariably presented as scientific fact despite growing uncertainty around these in the peer reviewed literature. The climate focussed storyline holds that forest biomass can be utilised to provide climate change mitigation benefit, but that this should be grounded in a holistic assessment of carbon balance, and governed via appropriate legislative controls to minimise the risk of harm to ecosystems and the functions they provide. Finally, the critical storyline rejects these claims that forest biomass utilisation can continue to provide the major part of European renewable energy. This storyline presents a narrative of the various ecological limits which forest biomass crosses, along with essentially all forms of bioenergy, as grounds to reject its utilisation from EU renewable policy support. These limits include physical impacts upon the climate, biodiversity, water and soils, and also less tangible influences on the status of forest ecosystems as natural or artificial.

The discourse coalition concept was more difficult to apply, potentially explained by the ongoing nature of the debate. While the forestry prioritised storyline 1 was found to be associated with a consistent grouping of forest and energy industry actors, a blurring was apparent between the other two storylines regarding their presentation of certain narrative elements. This occurred particularly in relation to the reported impacts of forest biomass upon community health, previously located solely in the critical third storyline. This narrative became included in more recent texts from actors associated with the climate focused storyline 2, resulting in a more critical discourse emerging here which does not easily link in with the storyline’s previous calls for forest biomass regulation within a revised renewable energy policy.

This fluidity between the discourses and actors associated with storylines 2 and 3, as well as the discursive stability displayed by the pro-biomass first storyline, is likely a result of the ongoing evolution of the renewable energy legislation as RED II approached its finalization in late 2018. Actors were continually reacting to intertextual and external factors and this was shaping their emerging discourses. While storyline 1 proponents were rallying behind their message that forest biomass based energy is a win-win for climate, environment and society, the others were scrambling to exert more influence on renewable energy policy, in part by adopting more critical narratives within their discourse.

It must be noted that the authors’ own choices have played a major role in shaping the various texts into the storylines presented here. The three storylines represent our own interpretation of how the forest biomass debate has developed in the decade leading up to the adoption of REDII. Three storylines are a manageable number, which certainly played a role in our decision making for the purposes of clarity and comparability. Several further storylines could have been split from the three presented here, particularly from storyline 2 in which a degree of variation was apparent in the risks or benefits associated with forest biomass.

That said, our interpretation of the gathered texts into these three storylines was guided by our research questions, particularly regarding the variable definitions of sustainability and nature. These interrelated concepts acted as lenses for the analysis, revealing much about the roots of the disagreements and contradictions within the biomass debate. The three storylines lie on a continuum regarding both of these interrelated concepts, as shown in Fig 3. The first storyline applies the most narrow sustainability definition, prioritising economic forest management, linking closely with an anthropogenic view of the forest as a resource, and stating that additional forest functions such as biodiversity value also require human intervention to obtain fully. The climate focussed storyline 2 applies a broader definition of sustainability prioritising an avoidance of environmental and climate change risk, within which the forest gains value as an entity in itself. Storyline 3 develops these concepts further, holding strongly biocentric interpretations of sustainability and nature, and a deep scepticism of resource management guided by economic rationale alone.

Despite the discursive differences created by these deeply held opposing views of what sustainability and nature *are* and what this means for forest biomass, there were several points where narrative elements overlapped to some extent as shown in Fig 3. These can (hopefully) provide some insight for developing a more constructive forest biomass debate. All storylines for example discuss the potential benefits which community ownership of small-scale bioenergy systems can provide. Establishing consensus here however is complicated however as a great deal of uncertainty continues to surround the policy making process. This makes it hard to establish what role forest biomass and bioenergy more widely should play for communities, as seen in the highly discordant responses emerging from all sides whenever a development in the RED II process is released [48, 81].

While the purpose of this study was not necessarily to provide recommendations for guiding a more constructive debate, several ambiguities and contradictions observed in the storylines also persist in the policy making process, which we believe is perpetuating disagreement. The issue of scale for example remains problematic as it is unclear exactly where, how much, and for how long forest biomass will continue to provide most of the European renewable energy. Related to this, the role of bioenergy within the emerging bioeconomy concept also raises questions regarding how natural resource consumption is conceptualised, for example regarding the balance between economic stability and climate change mitigation, and how these are prioritised over other ecosystem functions. Both issues would benefit from further study.

The utilisation of forest biomass for energy, along with many environmental issues, touches multiple parts of society. Here we argue that a large driver of the conflicts within the debate studied here is a broad disagreement regarding what relationship we as humans should have with forests. The ongoing debate around forest biomass policy at the EU level should primarily be concerned with establishing a workable renewable energy system, which actively mitigates climate change. Including forest biomass in renewable energy policy is complicated as a myriad of additional societal and ecological factors previously governed by appropriate policy regimes external to energy, have become necessarily included within the legislative text. This has generated substantial room for disagreement between diverse actors outside formal policymaking channels who seek to align the legislative outcome with their worldviews. Defining a clear role for scientific evidence or expert knowledge therefore becomes highly complicated in the forest biomass case, as there are an almost infinite number of knowledges, which can be considered, from ecology to human health, and actors prioritised these depending on their relevance to their arguments. This has clear implications for the inclusion of such evidence in the policymaking process, which must navigate and weigh the truth claims contained within the three storylines discussed here, alongside those of external discourses.

## Supporting information

S1 File Texts included in the discourse analysis. (DOCX)
