# Supplementary material for: Understanding the sustainability debate on forest biomass for energy in Europe: A discourse analysis
Source: PLoS One. 2021 Feb 17;16(2):e0246873. doi: 10.1371/journal.pone.0246873 (PMC7888638; doi:10.1371/journal.pone.0246873)
Supplement: S1 File — (DOCX) [file pone.0246873.s001.docx]

Source texts

ActionAid, BirdLife Europe and Central Asia, Climate Action Network Europe, European Environmental Bureau, Fern, Greenpeace, Oxfam, Transport & Environment, Wetlands International, and WWF. PITFALLS AND POTENTIALS THE ROLE OF BIOENERGY IN THE EU CLIMATE AND ENERGY POLICY POST 2020: NGO RECOMMENDATIONS. [Cited 20/09/2018] Available from <https://www.birdlife.org/sites/default/files/attachments/Bioenergy_post_2020_NGO%20recs.pdf>

AEBIOM. Biomass from sustainable forest: a carbon neutral source of renewable energy. European Biomass Association. [Cited 2019 Sept 15]. In: AEBIOM website [Internet]. URL no longer available.

AEBIOM. Sustainability criteria for solid and gaseous biomass. European Biomass Association. 2011 May 27 [Cited 2019 Sept 10]. In: European Biogas website [Internet]. Available from: <https://www.europeanbiogas.eu/wpcontent/uploads/2019/05/position_sustainability-criteria_2011.pdf>

AEBIOM. Sustainable Biomass Backs European Economy. European Biomass Association. 2014 Dec 3 [Cited 2019 Sept 15]. In: AEBIOM website [Internet]. URL no longer available.

AEBIOM. AEBIOM position on “A sustainable bioenergy policy for the period after 2020”. European Biomass Association. 2016 May 10. [Cited 2019 Sept 11]. In: agricultura.gencat.cat [Internet]. Available from: <http://agricultura.gencat.cat/web/.content/de_departament/de02_estadistiques_observatoris/27_butlletins/02_butlletins_nd/documents_nd/fitxers_estatics_nd/2016/0176_2016_ERenovables-Biomassa-prospectiva-Europa.pdf>

AEBIOM. AEBIOM first reaction to the Renewable Energy Directive vote in the European Parliament’s environmental committee. October 23, 2017 AEBIOM. European Biomass Association. [Cited 2019 Sept 10]. In: Bioenergy Europe [Internet]. Available from: <https://bioenergyeurope.org/article/135-aebiom-first-reaction-to-the-renewable-energy-directive-vote-in-the-european-parliament-s-environmental-committee.html>

AEBIOM. AEBIOM’S reaction to IEA Bioenergy response to Chatham House’s publication on Bioenergy climate impact. European Biomass Association. 2017 Mar 13 [Cited 2019 Sept 11]. In: Bioenergy Europe [Internet]. Available from: <https://bioenergyeurope.org/article/124-aebiom-s-reaction-to-iea-bioenergy-response-to-chatham-house-s-publication-on-bioenergy-climate-impact.html>

AEBIOM. Ahead of ENVI Committee’s vote on bioenergy sustainability, new insights revealed by AEBIOM’s latest Statistical Report. European Biomass Association. 2017 Oct 18 [Cited 2019 Sept 11]. In: Svebio.se [Internet]. Available from: <https://www.svebio.se/en/press/nyheter/ahead-envi-committees-vote-bioenergy-sustainability-new-insights-revealed-aebioms-latest-statistical-report/>

AEBIOM. The “Burning Issue” – When anti-bioenergy communication goes bad. European Biomass Association. 2017 Feb 19 [Cited 2019 Sept 11]. In: Bioenergy Europe [Internet]. Available from: <https://bioenergyeurope.org/article/122-when-anti-bioenergy-communication-goes-bad.html>

AEBIOM. The Eickhout paradox: How to reach 45% renewables by 2030 while jeopardising the EU’s main source of renewable energy?, European Biomass Association. 2017 June 8 [Cited 2019 Sept 12]. In: Bioenergy Europe [Internet]. Available from: <https://bioenergyeurope.org/component/attachments/attachments.html?id=234&task=download>

AEBIOM. Factsheet: Solid Bioenergy in Questions. European Biomass Association. 2017 June 1 [Cited 2019 Sept 10]. In: AEBIOM website [Internet]. URL no longer available.

AEBIOM. Smoke and mirrors prior to MEPs’ vote on REDII. European Biomass Association. 2018 Jan 11 [Cited 2019 Sept 12] In: Euractiv Blog [Internet]. Available from: <https://www.euractiv.com/section/energy/opinion/smoke-and-mirrors-prior-to-meps-vote-on-redii/>

AEBIOM. Untitled press release, 17.10.17. European Biomass Association. 2018 [Cited Sept 12]. In: AEBIOM website [Internet]. URL no longer available.

Beddington, J., S. Berry, K. Caldeira, W. Cramer, F. Creutzig, et al. EU must not burn the world's forests for 'renewable' energy. The Guardian. 2017 Dec 14 [Cited 2019 Sept 9]. Available from: <https://www.theguardian.com/environment/2017/dec/14/eu-must-not-burn-the-worlds-forests-for-renewable-energy#:~:text=The%20European%20Union%20is,current%20renewable%20energy%20by%202030>.

Beddington, J., S. Berry, K. Caldeira, W. Cramer, F. Creutzig, and others. Letter from Scientists to the EU Parliament regarding forest biomass. 2018 Jan 18 [Cited 2019 Sept 8]. In: Euractiv Blog [Internet]. Available from: <https://www.euractiv.com/wp-content/uploads/sites/2/2018/01/Letter-of-Scientists-on-Use-of-Forest-Biomass-for-Bioenergy-January-12-2018.pdf>

Biofuelwatch. BIOENERGY IN THE EUROPEAN COMMISSION’S PROPOSED POST-2020 EU RENEWABLE ENERGY DIRECTIVE: AN ANALYSIS OF PROPOSALS AND IMPACTS. 2016 Dec 26 [Cited 2019 Sept 15]. In: Biofuelwatch.org.uk [Internet] Available from: <https://www.biofuelwatch.org.uk/wp-content/uploads/red-briefing_final.pdf>

Biofuelwatch, and co-signatories. Large-scale bioenergy must be excluded from the EU's renewable energy definition. The Ecologist. 2016 Feb 10. Available from: <https://theecologist.org/2016/feb/10/large-scale-bioenergy-must-be-excluded-eus-renewable-energy-definition>

BirdLife, and Transport & Environment. How much sustainable biomass does Europe have in 2030? 2016 Nov 10. In: Transport & Environment Website [Internet]. Available from: <https://www.transportenvironment.org/publications/how-much-sustainable-biomass-does-europe-have-2030>

BirdLife, and Transport & Environment. Bioenergy: the ugly truth. BirdLife Europe and Central Asia. 2017 Dec 14 [Cited 2019 Sept 16]. BirdLife Europe and Central Asia Youtube channel [Internet]. Available from: <https://www.youtube.com/watch?v=A_7N2940w2Y&ab_channel=BirdLifeEuropeandCentralAsia>

BirdLife Europe, Fern, and Transport & Environment. Ensuring bioenergy comes clean in the Clean Energy Package. 2017 May 31 [Cited 2019 Sept 12]. In: Transport & Environment Website [Internet]. Available from: <https://www.transportenvironment.org/sites/te/files/publications/Position%20paper_Ensuring%20bioenergy%20comes%20clean%20in%20the%20Clean%20Energy%20Package%20May%202017.pdf>

Brack, D. Woody Biomass for Power and Heat: Impacts on the Global Climate. Chatham House. 2017 Feb 23 [Cited 2019 Sept 9]. In: Chatham House Website [Internet]. Available from: <https://www.chathamhouse.org/2017/02/woody-biomass-power-and-heat>

CEPF. Key ways to maximize potential of forest bioenergy to decarbonize Europe underlined. The Confederation of European Forest Owners. 2018 Jan 16 [Cited 2019 Sept 11]. In: EUSTAFOR website [Internet]. Available from: <https://eustafor.eu/key-ways-to-maximize-potential-of-forest-bioenergy-to-decarbonize-europe-underlined/>

Drax. 5 things you never knew about forests. 2016 Oct 6 [Cited 2019 Sept 8]. Drax website [Internet]. Available from: <https://www.drax.com/sustainability/5-things-you-never-knew-about-forests/>

Drax. Is biomass demand out of control?, Drax website. 2018 Apr 13 [Cited 2019 Sept 10]. In: Drax website [Internet]. Available from: <https://www.drax.com/sustainability/biomass-demand-control/>

Drax, and Robertsbridge. The biomass sustainability story. Drax. 2017 Dec 4 [Cited 2019 Sept 9]. In: Drax Youtube Channel [Internet]. Available from: <https://www.youtube.com/watch?v=Z8_Y3fpqlTI&ab_channel=Drax>

EASAC. Multi-functionality and sustainability in the European Union’s forests. 2017 May 11 [Cited 2019 Sept 12]. In: EASAC website [Internet]. Available from: <https://easac.eu/publications/details/multi-functionality-and-sustainability-in-the-european-unions-forests/>

EFI. Forest biomass, carbon neutrality and climate change mitigation. European Forestry Institute. 2016 Oct 12 [Cited 2019 Sept 11]. In: European Forestry Institute website [Internet]. Available from: <https://efi.int/publications-bank/forest-biomass-carbon-neutrality-and-climate-change-mitigation>

EUBIA. not dated. Biomass energy factsheet. [Cited 2019 Sept 15]. In: EUBIA website [Internet]. Available from: <https://www.eubia.org/cms/wiki-biomass/>

EUSTAFOR. Sustainable forestry enables the bioeconomy. 2017 Nov 17 [Cited 2019 Sept 16]. In: EUSTAFOR website [Internet]. Available from: <https://eustafor.eu/sustainable-forestry-enables-the-bioeconomy-eight-policy-messages-eustafor-looks-ahead-to-a-renewed-bioeconomy-strategy/#:~:text=Sustainably%20managed%20forests%20and%20wood,and%20highly%20energy%2Dintensive%20materials>.

EUSTAFOR, CEPF, COPA, COGECA, UEF, FECOF, and USSE. Sustainably managed forests are a proven source of sustainable biomass for bioenergy. 2017 Apr 4 [Cited 2019 Sept 7]. In: EUSTAFOR website [Internet]. Available from: <https://eustafor.eu/sustainably-managed-forests-are-a-proven-source-of-sustainable-biomass-for-bioenergy/>

Fern. Briefing note: Burning trees for energy is no solution to climate change. Brussels. 2016 Oct 14 [Cited 2019 Sept 7]. In: Fern website [Internet]. Available from: <https://www.fern.org/publications-insight/burning-trees-for-energy-is-no-solution-to-climate-change-340/>

Fern. A dangerous delusion: Debunking the myths around sustainable forests and the EU’s bioenergy policy. Brussels. 2016 Oct 19 [Cited 2019 Sept 8]. In: Fern website [Internet]. Available from: <https://www.fern.org/publications-insight/a-dangerous-delusion-debunking-the-myths-around-sustainable-forests-and-the-eus-bioenergy-policy-339/>

Fern. The European Parliament on climate: Blow hot, blow cold. Brussels. 2018 Feb 6 [Cited 2019 Sept 14]. In: Fern website [Internet]. Available from: <https://www.fern.org/es/recursos/the-european-parliament-on-climate-blow-hot-blow-cold-149/>

Fern. Fern’s analysis of the agreement on a new EU Renewable Energy Directive for the period after 2020. Brussels. 2018 Jun 14 [Cited 2019 Sept 16]. In: Fern website [Internet]. Available from: <https://www.fern.org/fileadmin/uploads/fern/Documents/Fern%20Analysis%20of%20REDII.pdf>

Fern, and M. Holland. Covered in smoke: Why burning wood threatens the health of Europeans. Brussels. 2018 Jan 5. In: Fern website [Internet]. Available from: <https://www.fern.org/publications-insight/covered-in-smoke-why-burning-biomass-threatens-european-health-160/#:~:text=Tens%20of%20thousands%20of%20EU,days%20lost%20to%20ill%20health>.

FoEE. Biomass ‘dirtier than coal’. 2012 Nov 12 [Cited 2019 Sept 5]. In: FOEE website [Internet]. Available from: <https://www.foeeurope.org/biomass-dirtier-than-coal-121112#:~:text=Burning%20whole%20trees%20in%20power,%2C%20the%20RSPB%2C%20and%20Greenpeace.&text=Burning%20biomass%20for%20energy%20is%20far%20from%20being%20carbon%20neutral>.

FoEE. 2014. World’s land and forests at threat from Europe’s demand for bioenergy.

FoEE, B. International, G. Europe, and and others. Open letter: NGOs call for suspension of biofuels targets. 2008 Jan 11 [Cited 2019 Sept 8]. In Transport & Environment website [Internet]. Available from: <https://www.transportenvironment.org/press/ngos-call-suspension-biofuels-targets>

Forest Europe, UNECE, and FAO. State of Europe’s Forests 2015. Status and Trends in Sustainable Forest Management in Europe. Ministerial Conference on the Protection of Forests in Europe, Forest Europe, Liaison Unit Madrid, Madrid. 2015 Dec 15 [Cited 2019 Sept 8]. In: Forest Europe website [Internet]. Available from: <https://foresteurope.org/state-europes-forests-2015-report/>

Global Forest Coalition, and Biofuelwatch. RED joint letter to MEPs. 2018 Jan 11 [Cited 2019 Sept 9]. In: Global Forest Coalition website [Internet]. Available from: <https://globalforestcoalition.org/campaigns/forests-trees-climate-change/page/7/>

Greenpeace. Make or break for renewable energy in Europe: Negotiations on EU renewables directive enter final stage. 2018 May 15 [Cited 2019 Sept 10]. In: Greenpeace website [Internet] Available from: <https://www.greenpeace.org/eu-unit/issues/climate-energy/1201/make-or-break-for-renewable-energy-in-europe/>

IEA Bioenergy. Response to Chatham House report “Woody Biomass for Power and Heat: Impacts on the Global Climate”. 2017 Mar 13 [Cited 2019 Sept 7]. In IEA bioenergy website [Internet]. Available from: <https://www.ieabioenergy.com/wp-content/uploads/2017/03/Chatham_House_response_supporting-doc.pdf>

IEEP. SUSTAINABLE BIOENERGY: An introduction to issues, IEEP work and the Way Forward. Institute for European Environmental Policy. Brussels, BE. 2016 [Cited 2019 Sept 8]. In: IEEP archive [Internet]. Available from: <https://ieep.eu/archive_uploads/2168/Work_on_bioenergy_-_new_template_format_final.pdf>

Juniper, T. Burning wood instead of coal in power stations makes sense if it's waste wood. The Guardian. 2017 Dec 19 [Cited 2019 Sept 7]. Available from: <https://www.theguardian.com/environment/2017/dec/19/burning-wood-instead-of-coal-in-power-stations-makes-sense-if-its-waste-wood#:~:text=2%20years%20old-,Burning%20wood%20instead%20of%20coal%20in%20power,sense%20if%20it's%20waste%20wood&text=Last%20week%2C%20a%20group,a%20solution%20to%20climate%20change>.

Juniper, T. Giving up coal. Drax website. 2017 Dec 7 [Cited 2019 Sept 8]. In: Drax website [Internet]. Available from: <https://www.drax.com/sustainability/giving-up-coal/>

Matthews, R., G. Hogan, and E. Mackie. Carbon impacts of biomass consumed in the EU: Supplementary analysis and interpretation for the European Climate Foundation. Forest Research. 2018 Nov 28 [Cited 2019 Sept 8]. In: Drax website [Internet]. Available from: <https://www.drax.com/wp-content/uploads/2019/10/CIB-Summary-report-for-ECF-v10.5-May-20181.pdf>

Matthews, R., L. Sokka, S. Soimakallio, N. Mortimer, J. Rix, M.-J. Schelhaas, T. Jenkins, G. Hogan, E. Mackie, A. Morris, and T. Randle. Review of literature on biogenic carbon and life cycle assessment of forest bioenergy. Forest Research. 2014 May 15 [Cited 2019 Sept 11]. Available from: <https://ec.europa.eu/energy/sites/ener/files/documents/2014_05_review_of_literature_on_biogenic_carbon_report.pdf>

NOAH, Biofuelwatch, Econexus, Global Forest Coalition, World Rainforest Movement, Rettet den Regenwald/Rainforest Rescue, and Corporate Europe Observatory. Bioenergy Out: Why bioenergy should not be included in the next EU Renewable Energy Directive. 2015 Sept 6 [Cited 2019 Sept 8]. In: Biofuelwatch website [Internet]. Available from: <https://www.google.com/search?q=Bioenergy+Out%3A+Why+bioenergy+should+not+be+included+in+the+next+EU+Renewable+Energy+Directive.&rlz=1C1CHBF_enDE819DE820&oq=Bioenergy+Out%3A+Why+bioenergy+should+not+be+included+in+the+next+EU+Renewable+Energy+Directive.&aqs=chrome..69i57j69i64.1055j0j4&sourceid=chrome&ie=UTF-8>

Rivers, M., and Drax. A working forest is like a carrot patch. Here’s why. 2018 Apr 11 [Cited 2019 Sept 9]. In Medium blog [Internet]. Available from: <https://medium.com/drax/a-working-forest-is-like-a-carrot-patch-heres-why-dd492f5ef560>

WWF. EU bioenergy policy. 2017 Jun 14 [Cited 2019 Sept 15]. In: WWF website [Internet]. Available from: <https://www.wwf.eu/?302612/EU-bioenergy-policy---position-paper>

WWF. EU Bioenergy Policy: Ensuring that the provisions on bioenergy in the recast EU Renewable Energy Directive deliver genuine climate benefits. 2017 Jun [Cited 2019 Sept 11]. In: WWF website [Internet]. Available from: <https://www.wwf.de/fileadmin/user_upload/EU_Bioenergy_Policy.pdf>
